# Cytosine Deaminase Base Editing to Restore *COL7A1* in Dystrophic Epidermolysis Bullosa Human: Murine Skin Model

**DOI:** 10.1016/j.xjidi.2023.100191

**Published:** 2023-02-19

**Authors:** Gaetano Naso, Soragia Athina Gkazi, Christos Georgiadis, Vignesh Jayarajan, Joanna Jacków, Roland Fleck, Leanne Allison, Olumide Kayode Ogunbiyi, John Alexander McGrath, Dusko Ilic, Wei-Li Di, Anastasia Petrova, Waseem Qasim

**Affiliations:** 1 Molecular and Cellular Immunology Unit, UCL Great Ormond Street Institute of Child Health, London, United Kingdom; 2 St John’s Institute of Dermatology, Kings College London, London, United Kingdom; 3 Centre for Ultrastructural Imaging, King’s College London, London, United Kingdom; 4 Department of Histopathology, Camelia Botnar Laboratories, UCL Great Ormond Street Institute of Child Health, London, United Kingdom; 5 Department of Women and Children’s Health, School of Life Course Sciences, Faculty of Life Sciences and Medicine, King’s College London, London, United Kingdom

## Abstract

Recessive dystrophic epidermolysis bullosa is a debilitating blistering skin disorder caused by loss-of-function mutations in *COL7A1,* which encodes type VII collagen, the main component of anchoring fibrils at the dermal−epidermal junction. Although conventional gene therapy approaches through viral vectors have been tested in preclinical and clinical trials, they are limited by transgene size constraints and only support unregulated gene expression. Genome editing could potentially overcome some of these limitations, and CRISPR/Cas9 has already been applied in research studies to restore *COL7A1* expression. The delivery of suitable repair templates for the repair of DNA cleaved by Cas9 is still a major challenge, and alternative base editing strategies may offer corrective solutions for certain mutations. We show highly targeted and efficient cytidine deamination and molecular correction of a defined recessive dystrophic epidermolysis bullosa mutation (c.425A>G), leading to restoration of full-length type VII collagen protein expression in primary human fibroblasts and induced pluripotent stem cells. Type VII collagen basement membrane expression and skin architecture were restored with de novo anchoring fibrils identified by electron microscopy in base-edited human recessive dystrophic epidermolysis bullosa grafts recovered from immunodeficient mice. The results show the potential and promise of emerging base editing technologies in tackling inherited disorders with well-defined single nucleotide mutations.

## Introduction

Recessive dystrophic epidermolysis bullosa (RDEB) is a severe genodermatosis caused by loss-of-function mutations in the *COL7A1* gene, which encodes for type VII collagen (C7) protein (Has et al., 2020). C7 is a key constituent of anchoring fibrils (AF) at the dermal−epidermal junction (DEJ), and its impairment compromises the integrity of the DEJ, leading to severe sublamina densa blistering and tissue cleavage (Burgeson, 1993). Currently, clinical management for RDEB is limited to supportive care, including daily dressings and meticulous wound care combined with nutritional supplements (Fine and Mellerio, 2009a, 2009b; Grocott et al., 2013).

Various therapeutic strategies have been investigated for the treatment of RDEB (Angelis et al., 2016; Hou et al., 2021; Naso and Petrova, 2020; Natsuga et al., 2021). These included intradermal (Remington et al., 2009; Woodley et al., 2004) and systemic (Woodley et al., 2013) injection of recombinant C7, intradermal injection of allogeneic fibroblasts (Petrof et al., 2013; Venugopal et al., 2013), hematopoietic cell transplantation (Tolar and Wagner, 2012; Wagner et al., 2010), and infusion of allogeneic MSCs (Conget et al., 2010; Petrof et al., 2015). Recently, topical application of a herpes simplex virus−derived vector encoding C7 has shown promise in clinical trials, although repeated applications were required (Gurevich et al., 2022).

In addition, several ex vivo gene therapy approaches using vector-modified fibroblasts or keratinocytes (KCs) have shown promising results in preclinical and clinical settings (Droz-Georget Lathion et al., 2015; Jacków et al., 2016; Latella et al., 2017; Lwin et al., 2019; Siprashvili et al., 2016), and no mutagenesis has been reported in these studies and trials to date.

However, genome editing strategies can be used to mediate precise, locus-specific correction of disease-causing mutations (Anzalone et al., 2020; Cox et al., 2015; Ran et al., 2013). The canonical CRISPR/Cas9 system relies on the introduction of double-stranded DNA breaks that are resolved through either nonhomologous end joining (NHEJ) or homology-directed repair. NHEJ typically produces small insertions and deletions (indels) and can be used to restore *COL7A1* expression through exon skipping and gene reframing (Bonafont et al., 2019; Kocher et al., 2020; Takashima et al., 2019). The homology-directed repair pathway, on the other hand, can be exploited to restore endogenous *COL7A1* sequence by introducing a donor template (Hainzl et al., 2017; Izmiryan et al., 2018; Jacków et al., 2019; Kocher et al., 2021; Webber et al., 2016). However, the low efficiency of this pathway in therapeutically relevant cells and the presence of accompanying NHEJ events and potentially deleterious indels often require antibiotic-resistance cassettes or single-cell selection to enrich for the corrected clones (Hainzl et al., 2017; Jacków et al., 2019; Webber et al., 2016).

In contrast, base editing tools involve double-stranded breaks-free site-specific modifications mediating either C−G to T−A (cytosine base editor [CBE]) or A−T to G−C (adenine base editor) conversions without double-stranded DNA cleavage or exogenous donor template (Gaudelli et al., 2017; Komor et al., 2017, 2016; Rees and Liu, 2018). In addition, base editing is able to correct single nucleotide mutations with sufficient efficiency without the need for a positive selection of gene-corrected cells. In dystrophic epidermolysis bullosa, approximately 76% of registered mutations are single nucleotide mutations (Naso and Petrova, 2019), and up to 61% of those can potentially be corrected with CBE or adenine base editor. Recently, adenine base editor−mediated base editing was successfully shown in primary RDEB fibroblasts and induced pluripotent stem cells (iPSCs) for two different *COL7A1* nonsense mutations (Osborn et al., 2020).

In this study, we investigated the potential of CBE-mediated correction of a known mutation in primary fibroblasts and patient-derived iPSCs. The splice-site mutation 425A>G, at exon 3 of *COL7A1* is a frequent mutation detected in various patient cohorts (Kern et al., 2006; Murata et al., 2004). We used third-generation human codon-optimized base editor *CBE3* mRNA and single-guide RNA (sgRNA) to target this pathogenic mutation. Efficient and specific nucleotide correction in patient iPSCs and primary fibroblasts was observed, leading to the restoration of C7 expression in vitro. Crucially, base-edited fibroblasts were able to restore the DEJ integrity by forming de novo AFs in a human:murine chimeric skin graft mouse model in vivo*.*

## Results

### Efficient base conversion in primary RDEB fibroblasts and iPSCs

**Figure 1:**
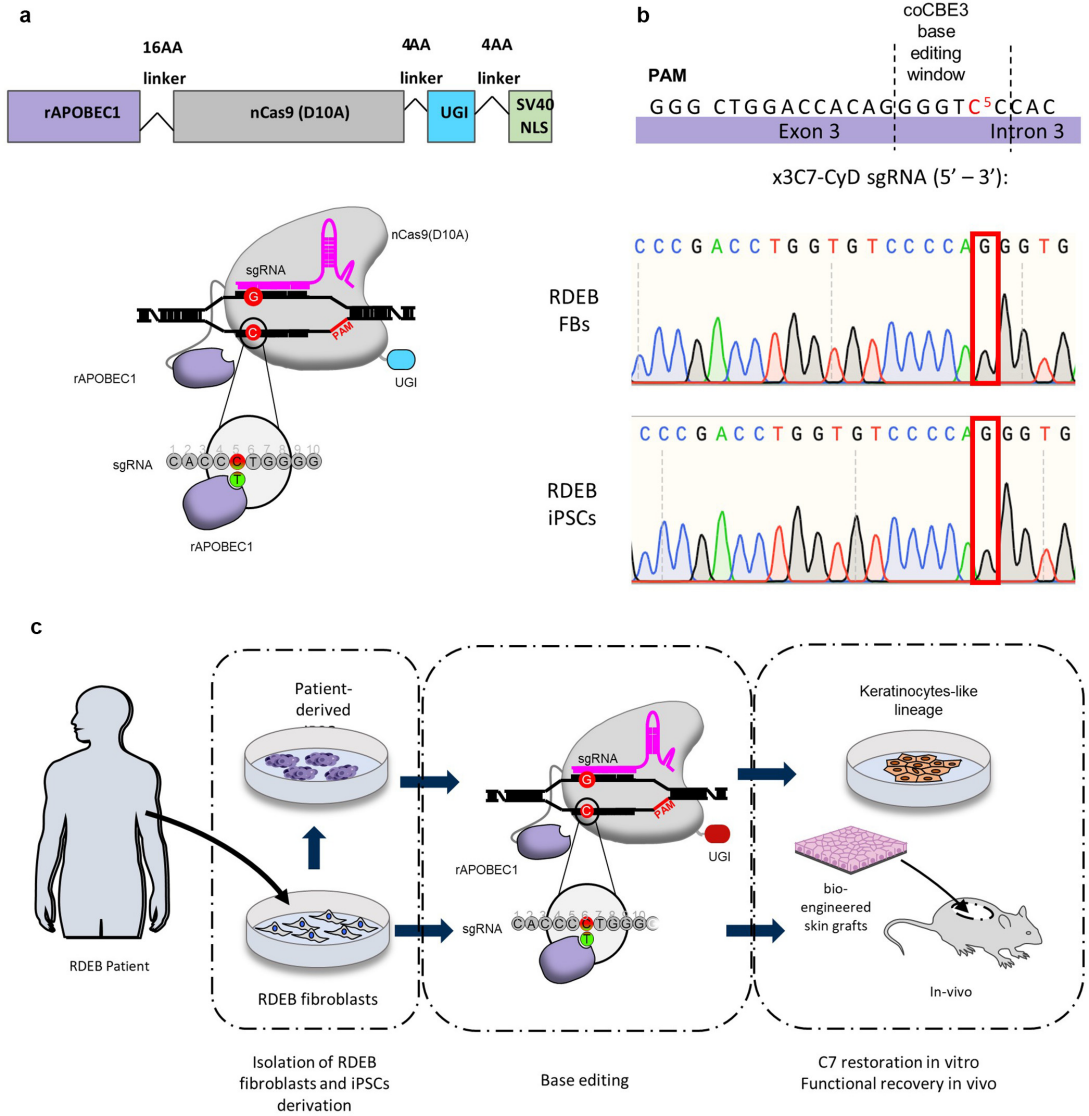
**Experimental design for cytosine base editing for the correction of a *COL7A1* mutation.** (**a**) Schematic of codon optimized cytosine base editor (coCBE3) protein structure and mechanism of action. Rat apolipoprotein B mRNA editing enzyme (rAPOBEC1) links to *Streptococcus pyogenes* Cas9 D10A nickase (nCas9) and a uracil glycosylase inhibitor (UGI) domain to prevent excision and reversion of U:G mismatches. After Cas9 binding, rAPOBEC1 mediates C-to-U conversion by deamination of single-stranded DNA displaced by the protospacer within a 5-bp editing window corresponding to the fourth and eighth nucleotides 5´ of the protospacer. (**b**) Schematic showing the x3C7-CyD guide RNA designed to target the exon3/intron3 junction of *COL7A1* at position C5 (antisense), corresponding to the c.425A>G RDEB point mutation highlighted in red. The dotted lines show the coCBE3 activity window. Below are Sanger-sequencing traces confirming the presence of homozygous c.425A>G mutation in human fibroblasts (top) and iPSCs (bottom). (**c**) RDEB fibroblasts were isolated and reprogrammed into iPSC. Both cell types were then gene edited by electroporation of *coCBE3* mRNA and x3C7-CyD sgRNA. Functional C7 recovery from base-edited fibroblasts was assessed in vivo using a human:murine xenograft skin model. Corrected iPSCs were differentiated toward keratinocyte-like cells to assess C7 restoration in vitro. C7, type 7 collagen; FB, fibroblast; iPSC, induced pluripotent stem cell; NLS, nuclear localization signal; RDEB, recessive dystrophic epidermolysis bullosa; sgRNA, single guide RNA.

**Figure 2:**
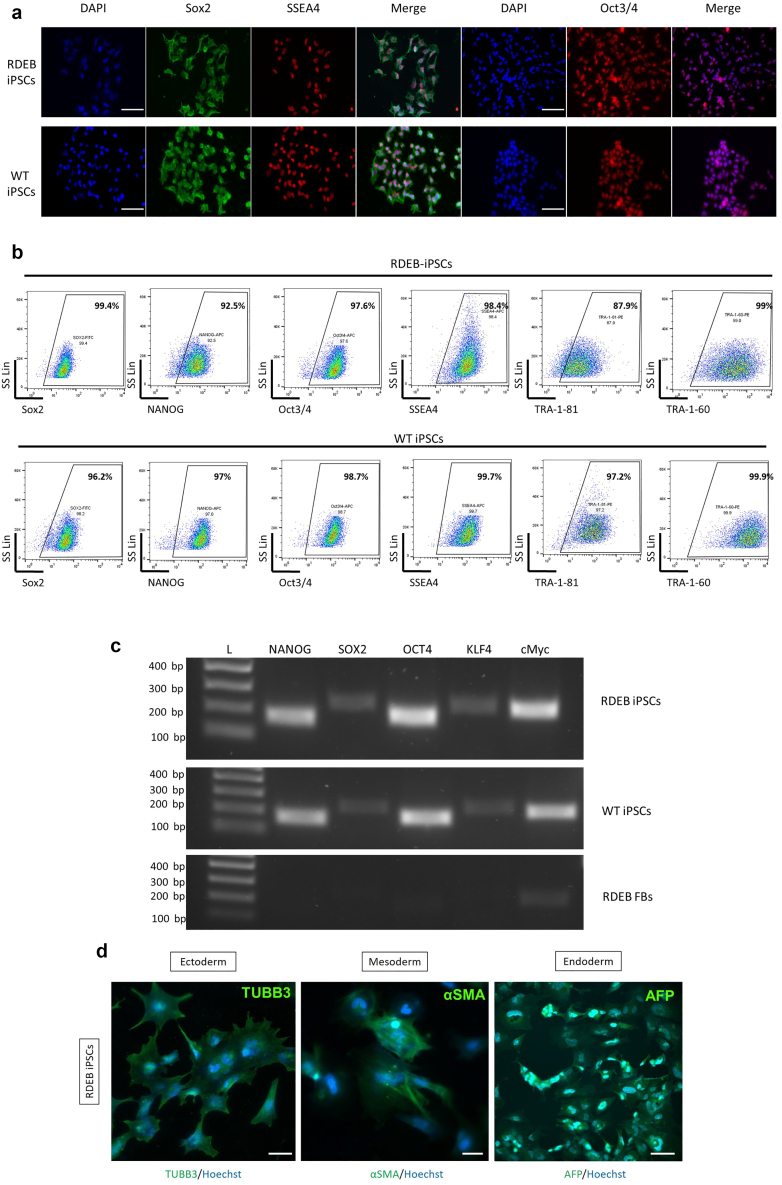
**Characterization of patient-derived RDEB iPSCs.** (**a**) In situ immunofluorescence staining for pluripotency markers. From left to right: Sox2, SSEA-4, and Oct3/4. Nuclei were counterstained with DAPI (blue). WT iPSCs were used as a positive control. Bar = 50μm. (**b**) Representative quantification of pluripotency-associated markers by flow cytometry for RDEB (top) and WT (bottom) iPSCs. (**c**) Confirmation of the expression of *NANOG*, *Sox2*, *Oct4*, *KFL4*, and *cMYC* pluripotency markers in RDEB (top) and WT (middle) iPSCs by RT-PCR. RDEB fibroblasts were used as a negative control (bottom). (**d**) RDEB iPSCs are able to differentiate toward all three germ layers as shown by their expression of TUBB3 (ectoderm), αSMA (mesoderm), and AFP (endoderm) in the trilineage differentiation assay. αSMA, α-smooth muscle actin; AFP, alpha-fetoprotein; iPSC, induced pluripotent stem cell; RDEB, recessive dystrophic epidermolysis bullosa; WT, wild type.

mRNA for the *CBE3* base editor was synthesized from a plasmid containing coCBE3 (Figure 1a). A specific sgRNA (×3C7-CyD) was designed so that the c.425A>G mutation is optimally located within the 5 bp CBE3 editing window at position C5 (Figure 1b). Sanger sequencing was used to confirm the presence of the c.425A>G mutation hotspot in both primary fibroblasts and iPSCs (Figure 1b). Codon-optimised CBE3 (*CoCBE3*) mRNA and sgRNA were delivered into primary fibroblasts and iPSCs generated from the patient’s cells harboring a homozygous c.425A>G mutation in *COL7A1* by electroporation. Patient iPSCs were differentiated into KC-like lineages to assess protein restoration in vitro, whereas fibroblasts were used to assess functional recovery in vivo (Figure 1c)*.* Patient-derived iPSCs expressed the markers of pluripotency as assessed by immunofluorescence, flow cytometry and RT-PCR, and were able to differentiate toward all the three germ layers in a trilineage differentiation assay (Figure 2).

**Figure 3:**
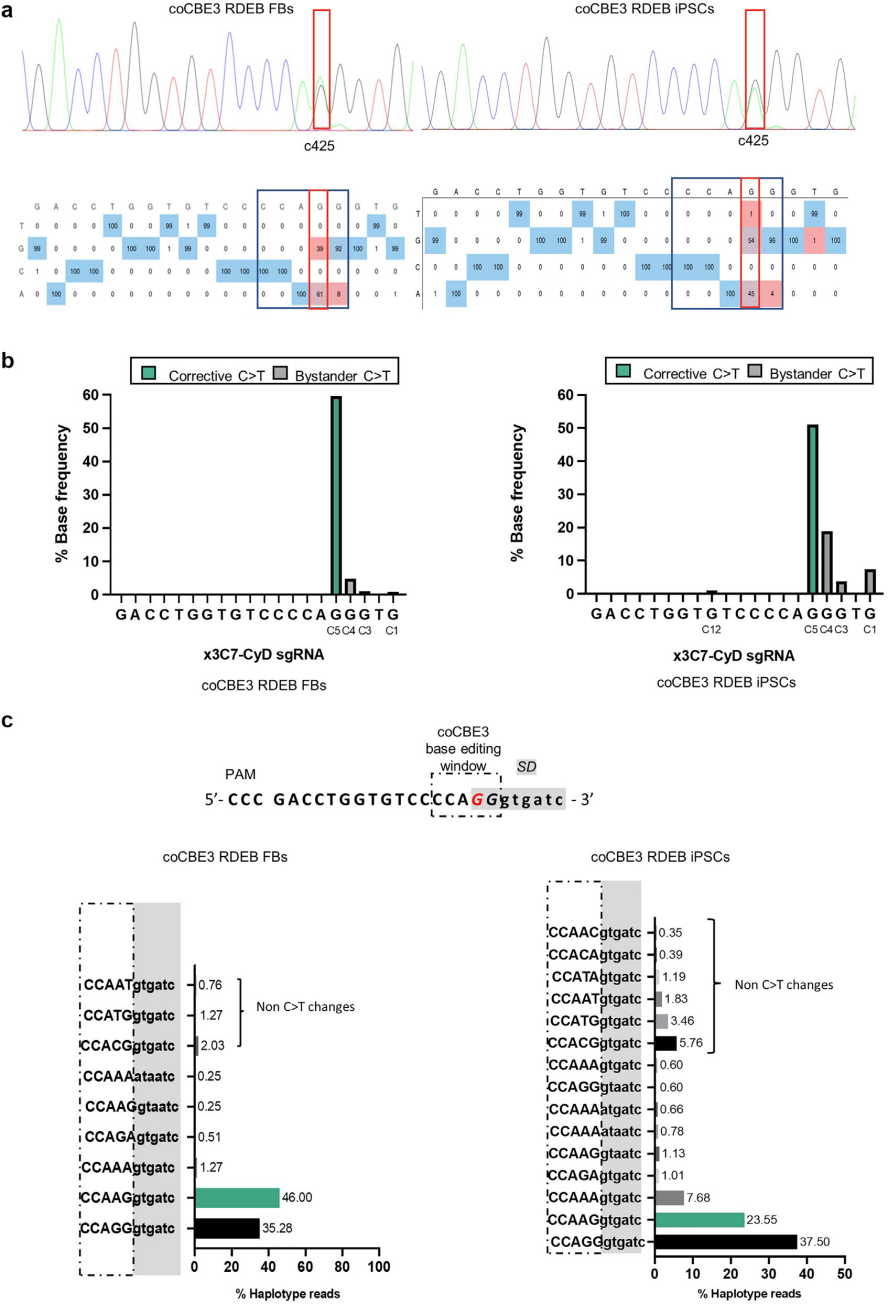
**Confirmation of efficient base editing in patient fibroblasts and iPSCs.** (**a**) Sanger sequencing and EditR analysis of base-edited RDEB fibroblasts (left) and iPSCs (right). The base editing window is boxed in blue, and the desired base change at position C5 (c.425) is boxed in red. Histogram legend: black, G; green, A; blue, C; red, T. The substitution rate at each position of the x3C7-CyD protospacer is shown in the table. A total of 61 and 45% of targeted C>T (G>A) conversion were detected in patient fibroblasts and iPSCs, respectively. Bystander C>T conversions at position C4 were detected in 8 and 4% of patient fibroblasts and iPSCs, respectively. (**b**) NGS analysis of coCBE3 activity in patient fibroblasts (left) and iPSCs (right) shows C>T conversion across the protospacer with the C positions indicated below. A total of 59.6 and 51% of the targeted c.425A>G mutation correction was detected in patient iPSCs and fibroblasts, respectively (green bar). On-target bystander C>T conversions were detected at position C4 (4.82 and 19.39% in patient fibroblasts and iPSCs, respectively) and outside the base-editing window at position C3 (3.89 and 1.01 and 3.89% in patient fibroblasts and iPSCs, respectively), C1 (7.44% in patient iPSCs only), and C12 (1.03% in patient iPSCs only). (**c**) CRISPResso2-based haplotype quantification of NGS data in base-edited fibroblasts (left) and iPSCs (right). The percentage of reads with a correction at position c.425 alone is shown by the green bar (46 and 23.55% in patient fibroblasts and iPSCs, respectively). Bystander C>T edits and non-C>T base changes are shown within the 5-bp coCBE3 deamination activity window in exon 3 of *COL7A1* (upper case letters, dotted box) and outside the base-editing window within the exon 3 SD sequence of *COL7A1* (lower case letters, highlighted in gray). iPSC, induced pluripotent stem cell; NGS, next-generation sequencing; PAM, protospacer adjacent motif; RDEB, recessive dystrophic epidermolysis bullosa; SD, splicing donor; sgRNA, single-guide RNA.

Sanger sequencing−based EditR analysis of the DNA from the treated cells revealed up to 61 and 45% of targeted C>T (G>A) base conversion at the desired c.425 (C5) position in patient fibroblasts and iPSCs, respectively (Figure 3a). Bystander C>T conversion at position c.426 (C4) was detected in 8 and 4% of Sanger sequencing reads in patient fibroblasts and iPSCs, respectively. The frequencies detected by Sanger sequencing analysis were further corroborated by on-target next-generation sequencing (NGS) analysis. On-target C>T conversion at position c.425/C5 was confirmed in over 51 and 59% of the reads for base-edited iPSCs and fibroblasts, respectively (Figure 3b). As initially observed by Sanger sequencing, NGS confirmed the presence of additional bystander on-target C>T conversion within the predicted coCBE3 editing window at position C4 (19.4% in iPSCs and 4.8% in fibroblasts) and outside the window at position C3 (3.9% and 1% in iPSCs and fibroblasts, respectively), C1 (7.4% in iPSCs only), and C12 (1% in patient iPSCs only). In addition, a small frequency of noncanonical C>T conversions was also observed at the target c.425 site (5.5% C>A and 6.6% C>G in iPSCs and 2.2% C>A and 2.7% C>G in fibroblasts). In total, changes within the sequencing window other than the desired C5 conversion totaled 42.9% for iPSCs and 11.7% for fibroblasts, indicating the need for further improvements.

To determine the frequency of C>T correction at position c.425 alone, haplotype-based analysis of the NGS data was carried out by CRISPResso2 (Clement et al., 2019) and revealed that up to 23.5 and 46% of reads harbored C>T changes at position c.425 alone without the presence of unwanted bystander effects in patient iPSCs and fibroblasts, respectively (Figure 3c).

To exclude NHEJ effects due to the possible residual nicking activity of the nCas9(D10A) within coCBE3 editing window (Komor et al., 2016), the presence of indels was evaluated by NGS. A small percentage of indels (3.6%; 2.5% deletions + 1.1% insertions) were detected in the target *COL7A1* sequence recognized by ×3C7-CyD sgRNA.

### Base editing resulted in a very low frequency of off-target guide-dependent events

**Figure 4:**
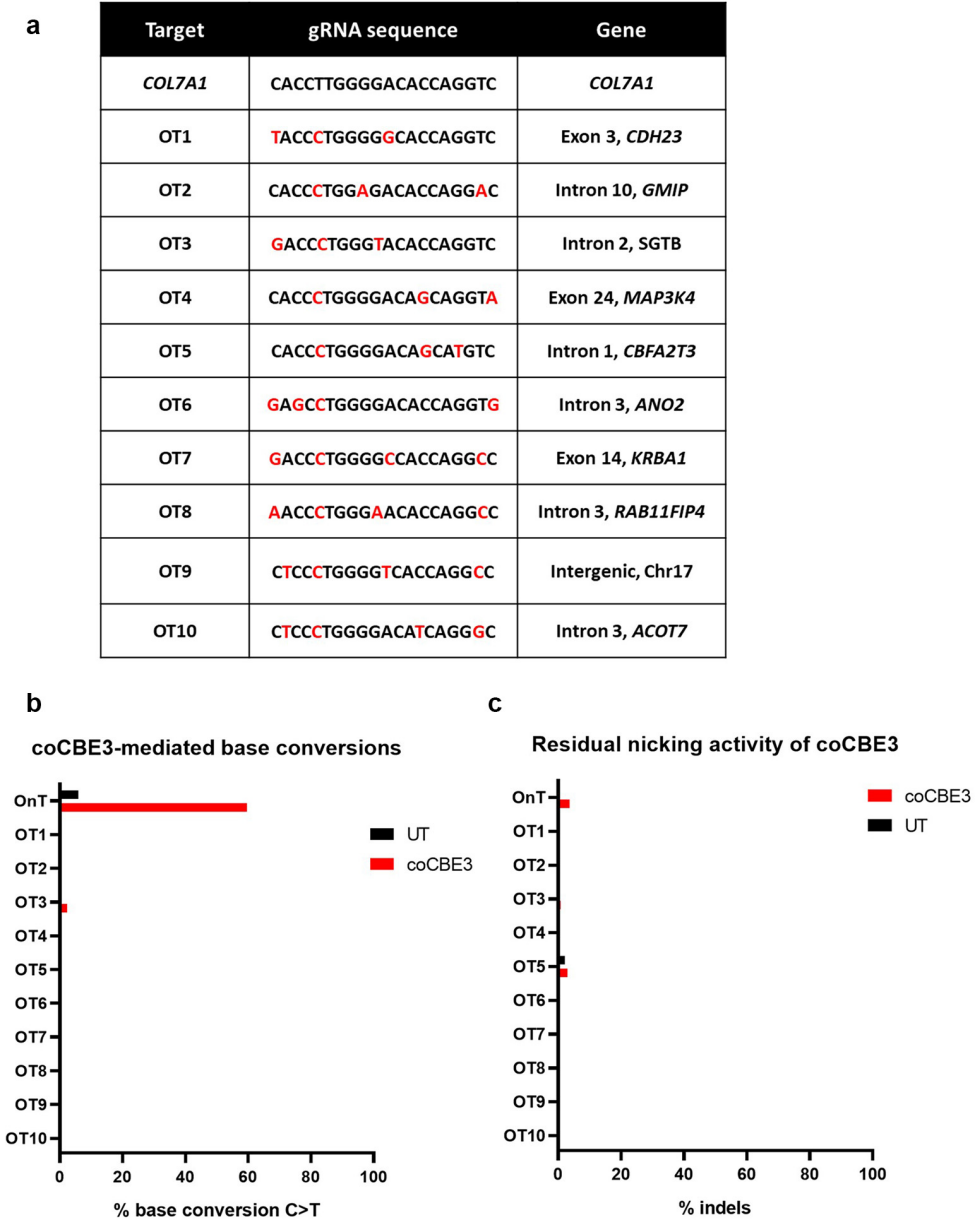
**Targeted base editing of a *COL7A1* mutation and potential guide-directed off-target sites.** (**a**) The top 10 off-target sites for x3C7-CyD sgRNA predicted by Benchling are shown in column 1. Highlighted in red are mismatch bases between the off-target site and the *COL7A1* x3C7-CyD sgRNA target (column 2). For each predicted off-target, the gene name and relative location within coding or noncoding sites are indicated in column 3. (**b**) A plot showing coCBE3-mediated C>T conversions within the base-editing window of *COL7A1* and off-target sites for untreated (UT) and treated samples (coCBE3). (**c**) A plot showing the percentages of indels for *COL7A1* and off-target sites for UT and treated samples (coCBE3). gRNA, guide RNA; indel, insertion and deletion; sgRNA, single-guide RNA.

To assess whether coCBE3 created off-target C>T editing in a guide-dependent fashion, the Benchling in silico predictive algorithm was used to identify the off-target regions that could potentially be targeted by the ×3C7-CyD sgRNA protospacer (Figure 4a). The top 10 identified off-target genomic loci were interrogated by NGS, and off-target edits within the coCBE3 editing window were detected at frequencies below 0.5% in 9 of 10 off-target sites (Figure 4b). A 4% C>T change was detected at position 5 of the base editing window in 1 of 10 evaluated sites (OT3) but was also present in untreated samples (*P* = 0.25) and therefore not attributed to base editing effects. A full list of C>T changes detected in the predicted off-target sites is detailed in Table 1.

**Table 1:** Next-Generation Sequencing Data Analysis of Top 10 Guide-Dependent Off-Target Sites

Gene	C Position	% Base Conversion	
		UT	CBE
*C7 ON*	1	0.00	0.78
	3	0.18	1.01
	**4**	**0.45**	**4.08**
	**5**	**5.82**	**59.62**
	12	0.00	0.15
	14	0.00	0.1
	15	0.09	0.15
	20	0.09	0.05
*C7 OT1*	3	0.02	0.07
	**4**	**0.05**	**0.00**
	**5**	**0.04**	**0.01**
	12	0.00	0.01
	14	0.05	0.05
	15	0.09	0.02
	20	0.03	0.01
*C7 OT2*	1	0.08	0.09
	3	0.09	0.05
	**4**	**0.06**	**0.09**
	**5**	**0.03**	**0.07**
	12	0.08	0.04
	14	0.05	0.07
	15	0.07	0.04
	20	0.02	0.00
*C7 OT3*	3	0.00	0.02
	**4**	**0.00**	**0.49**
	**5**	**0.10**	**4.09**
	12	0.05	0.19
	14	0.00	0.02
	15	0.05	0.00
	20	0.20	0.02
*C7 OT4*	1	0.00	0.00
	3	0.00	0.00
	**4**	**0.01**	**0.00**
	**5**	**0.00**	**0.00**
	12	0.00	0.01
	15	0.03	0.00
*C7 OT5*	1	0.15	0.18
	3	0.10	0.06
	**4**	**0.11**	**0.12**
	**5**	**0.02**	**0.02**
	12	0.00	0.06
	15	0.00	0.05
	20	0.09	0.00
*C7 OT6*	**4**	**0.13**	**0.15**
	**5**	**0.00**	**0.03**
	12	0.00	0.00
	14	0.00	0.00
	15	0.03	0.06
*C7 OT7*	3	0.09	0.00
	**4**	**0.09**	**0.00**
	**5**	**0.00**	**0.21**
	11	0.00	0.00
	12	0.05	0.00
	14	0.00	0.00
	15	0.10	0.00
	19	0.10	0.00
	20	0.00	0.11
*C7 OT8*	3	0.01	0.04
	**4**	**0.00**	**0.00**
	**5**	**0.00**	**0.00**
	12	0.03	0.00
	14	0.03	0.00
	15	0.03	0.00
	19	0.04	0.01
	20	0.04	0.07
*C7 OT9*	1	0.06	0.00
	3	0.00	0.00
	**4**	**0.00**	**0.00**
	**5**	**0.06**	**0.00**
	12	0.06	0.00
	14	0.00	0.00
	15	0.00	0.00
	19	0.00	0.21
	20	0.06	0.00
*C7 OT10*	1	0.03	0.02
	3	0.00	0.06
	**4**	**0.02**	**0.00**
	**5**	**0.00**	**0.04**
	12	0.03	0.05
	15	0.02	0.02
	20	0.00	0.03

Abbreviations: CBE, cytosine base edited sample; UT, untreated sample.

Percentage C>T conversion in CBE3 or UT samples across C bases within each 20 bp off-target sequence is shown. CBE3-activity window is highlighted in bold.

With respect to possible NHEJ effects due to possible residual nicking activity of the nCas9(D10A) within coCBE3, no significant NHEJ activity was detected in the off-target sites when compared with that in untreated samples (Figure 4c).

### Base editing restores full-length C7 expression in primary RDEB fibroblasts and iPSC-derived KC-like cells

**Figure 5:**
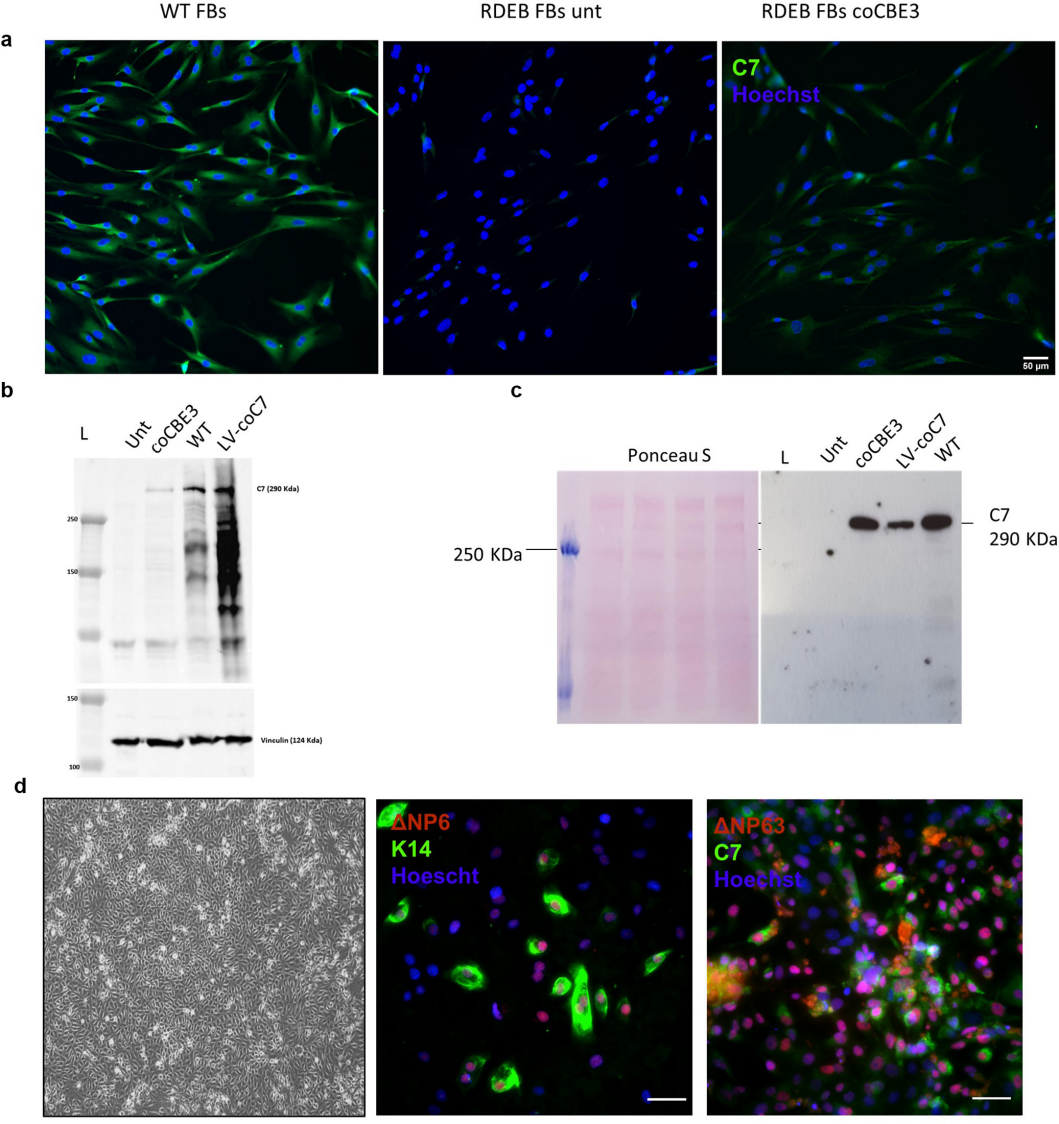
**Base editing restores C7 expression in vitro in fibroblasts and iPSC-derived keratinocyte-like cells.** (**a**) Restoration of C7 (green) expression in base-edited fibroblasts by immunofluorescence staining. Untreated RDEB fibroblasts and WT fibroblasts were used as negative and positive controls, respectively. Bar = 50 μm. (**b**) C7 western blotting from cell lysate confirms the presence of full-length (∼290 kDa) C7 in base-edited fibroblasts. No C7 expression was observed in untransduced cells. Lenti-C7−transduced fibroblasts and WT fibroblasts were used as positive controls. Vinculin was used as a loading control. (**c**) C7 western blotting using cell culture supernatant confirms that base-edited fibroblasts are able to secrete functional, full-length C7. No secreted C7 was detected in untreated RDEB fibroblasts. Lenti-C7−transduced fibroblasts and WT fibroblasts were used as positive controls. Ponceau S staining was used as a loading control. (**d**) Left: phase contrast image of base-edited iPSC-derived keratinocyte-like cells showing typical epidermal cell morphology. Middle: iPSC-derived keratinocyte-like cells coexpressing epidermal stem cell markers, △Np63 (red), and K14 (green). Right: iPSC-derived keratinocyte-like cells express de novo C7 (green). △Np63 expression is shown in red. Bar = 50 μm. RDEB FBs unt denotes untreated RDEB fibroblasts, RDEB coCBE3 FBs denotes base-edited fibroblasts, WT FBs denotes WT fibroblasts, LV-coC7 denotes RDEB fibroblasts transduced with lentiviral vector containing codon-optimized *COL7A1*, and L denotes ladder. C7, type 7 collagen; FB, fibroblast; iPSC, induced pluripotent stem cell; K14, keratin 14; RDEB, recessive dystrophic epidermolysis bullosa; WT, wild type.

C7 expression in coCBE3-edited RDEB fibroblasts was examined by immunostaining and immunoblotting. Positive C7 expression was detected in base-edited cells but not in untreated patient cells (Figure 5a). Immunoblotting results showed that the presence of 290 kDa band in base-edited fibroblasts corresponded to full-length C7 protein in total cell lysate (Figure 5b). Furthermore, immunoblotting detected a full-length C7 in the cell culture supernatant harvested from base-edited RDEB fibroblasts, indicating successful secretion of the protein (Figure 5c). Untreated patient cells and wild-type (WT) fibroblasts were used as negative and positive controls of C7 expression, respectively. RDEB fibroblasts transduced with the lentiviral vector containing the full-length codon-optimized *COL7A1* cDNA were used as an additional positive control (Georgiadis et al., 2016). To confirm the restoration of C7 in coCBE3-edited iPSCs, the cells were differentiated into KC-like cells using a previously described protocol (Petrova et al., 2014). Immunofluorescent analysis confirmed the restoration of C7 expression in approximately 29.4% of the base-edited cells (Figure 5d). Importantly, iPSC-derived KC-like cells displayed typical epidermal morphology and expression of epidermal stem cell markers, ΔNP63 and keratin 14.

### Base-edited fibroblasts restore skin integrity in human:murine skin grafts

To determine whether base-edited cells could result in the deposition and incorporation of C7 into the DEJ, a human:murine xenograft skin model was adopted (Di et al., 2012, 2011; Larcher et al., 2007). Bioengineered skin grafts generated by base-edited fibroblasts and untreated RDEB KCs were grafted on NSG mice. Bioengineered skin grafts incorporating untreated RDEB or healthy KCs and fibroblasts were used as negative and positive controls, respectively.

**Figure 6:**
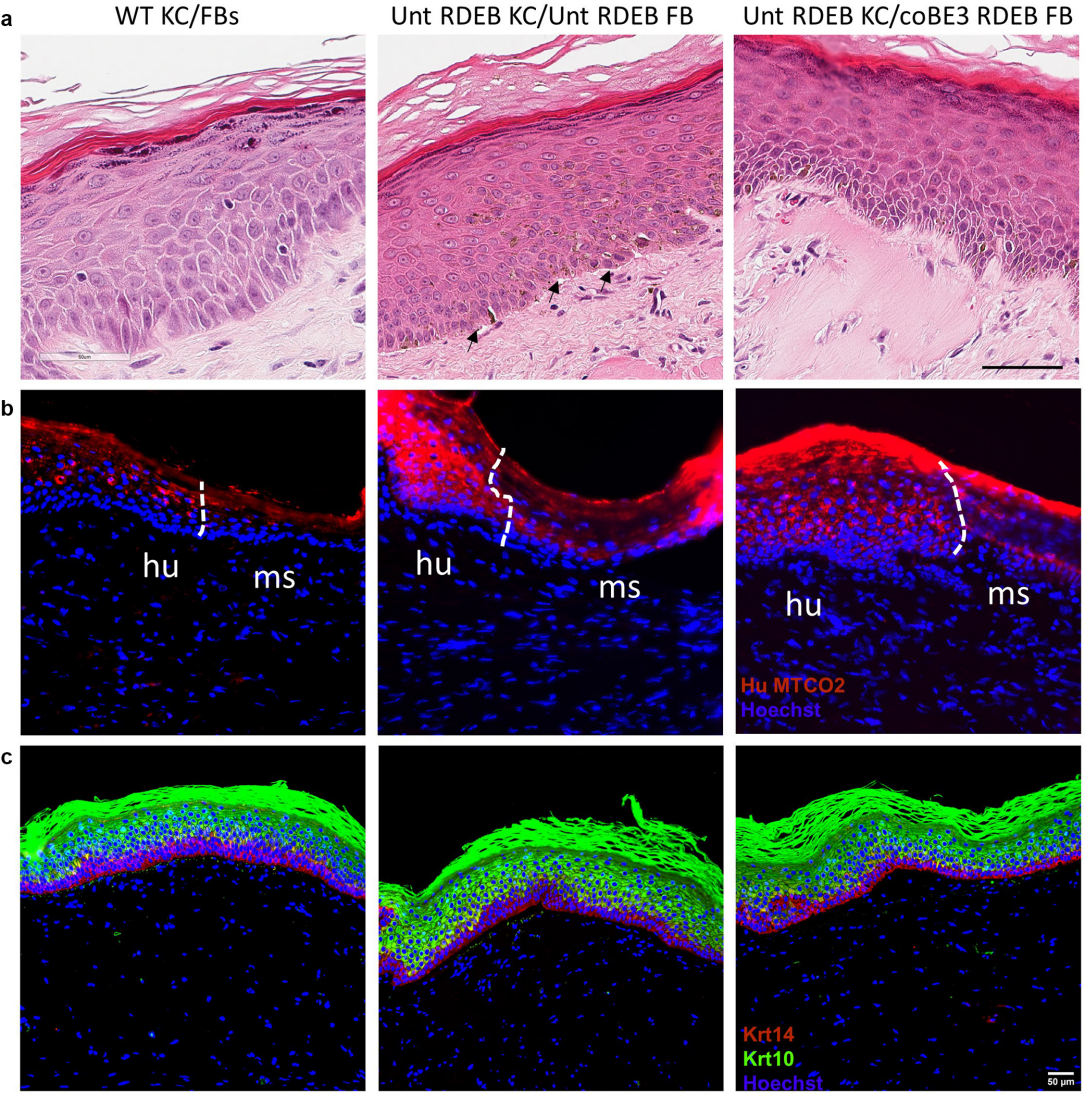
**Human skin equivalents produced using base-edited fibroblasts display normal epidermal morphology and stratification.** (**a**) H&E staining shows normal morphology of human skin. Blistering is shown by the black arrow. Bar = 60 μm. (**b**) Human origin of the graft was confirmed by human-specific Cytochrome C oxidase (complex IV) subunit II (MTCO2) staining (red). A white dotted line demarcates the border between mouse and human tissue. (**c**) Immunofluorescent staining for keratins 14 (red) and 10 (green) showed a normal differentiation pattern of the grafts, with a basal expression of the former and suprabasal localization of the latter. Bar = 50 μm. WT KCs/FBs denotes wild-type grafts, Unt RDEB KC/Unt RDEB FB denotes untreated RDEB grafts, Unt RDEB KC/coCBE RDEB FB denotes grafts containing base edited fibroblasts and untreated RDEB keratinocytes, hu denotes human, and ms denotes mouse. FB, fibroblast; KC, keratinocyte; RDEB, recessive dystrophic epidermolysis bullosa.

Upon harvesting, the morphology of the grafts was evaluated by H&E staining, which revealed multiple stratified epidermal layers in all conditions (Figure 6a). Blistering and splitting at the DEJ were observed in the grafts generated using untreated RDEB cells, which closely resembled the human disease phenotype. On the contrary, no blistering was detected in the grafts generated using base-edited fibroblasts or WT fibroblasts. The human origin of the grafted area was confirmed by species-specific staining for mitochondrial marker (complex IV subunit II) to demarcate human:murine borders (Figure 6b). All grafts showed normal distributions of keratins 14 and 10 in the basal and suprabasal epidermal layers, respectively (Figure 6c).

### Base-edited fibroblasts deposit C7 at the DEJ and form de novo AFs in vivo

**Figure 7:**
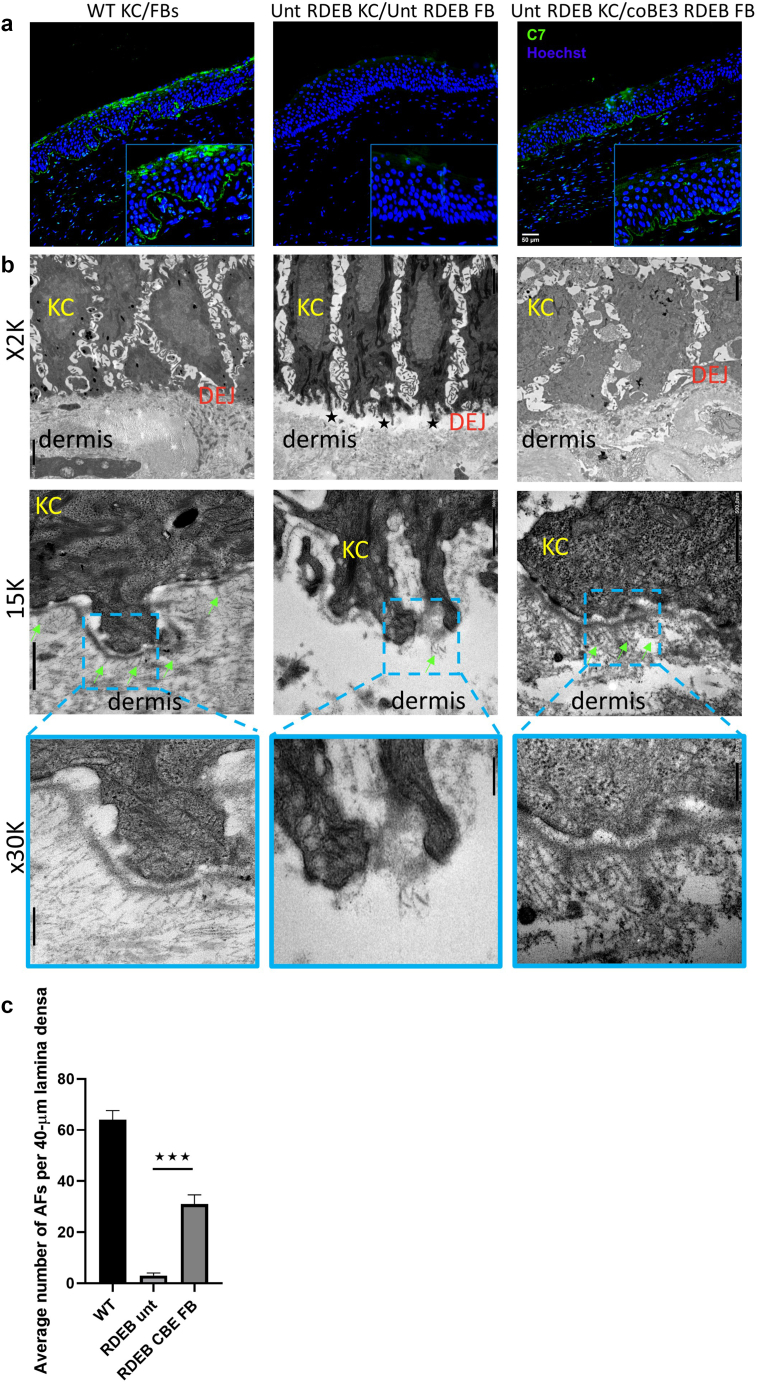
**In vivo functional correction through type VII collagen deposition and de novo AF formation.** (**a**) Immunofluorescent analysis of C7 (green) expression at the DEJ. Strong C7 expression can be seen throughout the DEJ of WT grafts (left), whereas it is completely absent in RDEB grafts (middle). Robust, albeit patchy, C7 expression can be detected in grafts containing base-edited fibroblasts. Inserts show a magnified view of the DEJ. Bar = 50 μm. (**b**) TEM analysis of the skin grafts shows the formation of de novo AFs. Images are shown at the following magnifications: ×2,000, bar = 2 μm; ×15,000, bar = 500 nm; and ×30,000, bar = 200 nm. Green arrows point at AFs. Black stars show blisters. (**c**) Quantitative analysis revealed a significantly higher density of AFs in grafts containing base-edited fibroblasts compared with that in untreated RDEB grafts. Statistical analysis was carried out using the Student’s *t*-test. Error bars represent the SD; n = 3 for each condition. WT KC/FBs denotes wild-type grafts, Unt RDEB KC/Unt RDEB FB denotes untreated RDEB grafts, and Unt RDEB KC/coCBE RDEB FB denotes grafts containing base-edited fibroblasts and untreated RDEB keratinocytes. AF, anchoring fibril; C7, type 7 collagen; DEJ, dermal−epidermal junction; FB, fibroblast; KC, keratinocyte; RDEB, recessive dystrophic epidermolysis bullosa; TEM, transmission electron microscopy.

**Figure 8:**
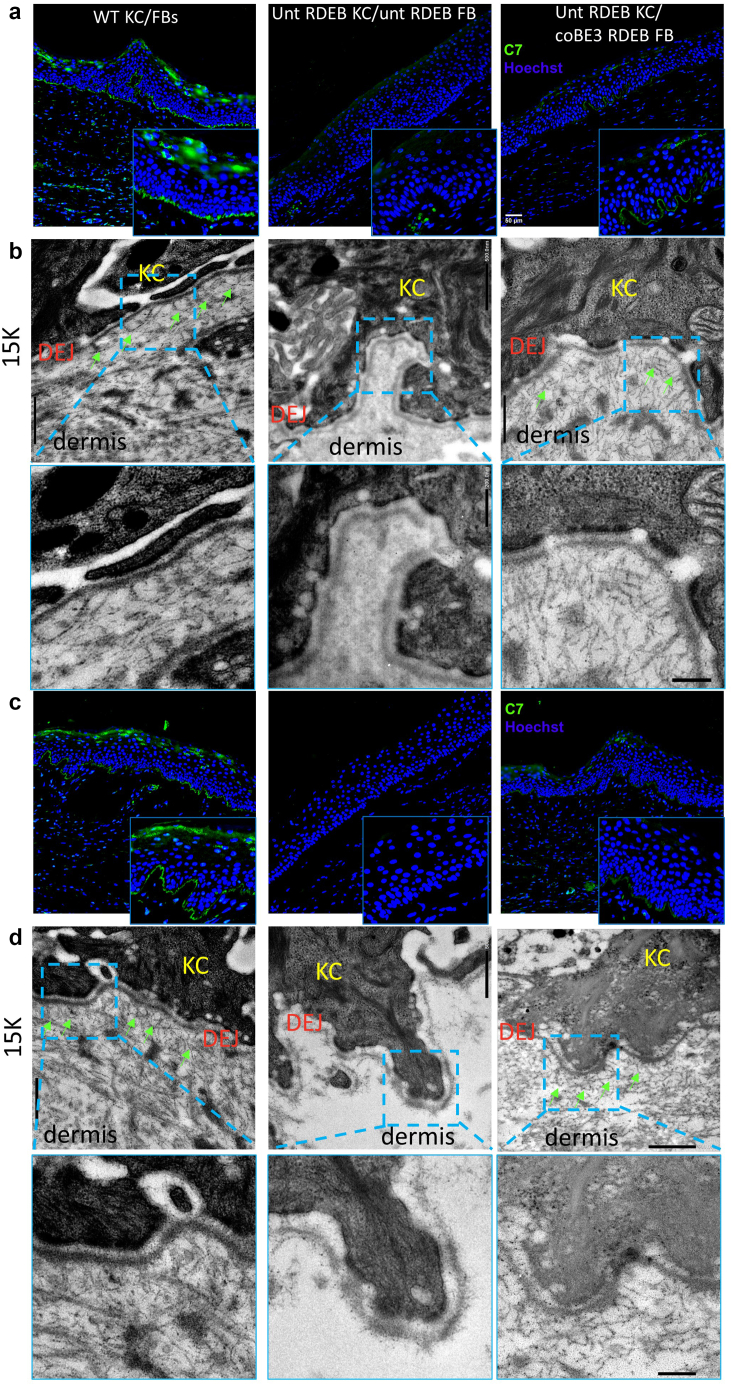
**Representative images of C7 expression by immunofluorescence and AF formation by TEM.** (**a, c**) Immunofluorescent analysis of C7 (green) expression at the DEJ. Strong C7 expression can be seen throughout the DEJ of WT grafts (left), whereas it is completely absent in RDEB grafts (middle). Robust, albeit (**a**) patchy or continuous (**c**) dim C7 expression can be detected in the grafts containing base-edited fibroblasts. Inserts show a magnified view of the DEJ. Bar =50 μm. (**b, d**) Representative TEM images of the grafts shown in **a** and **c.** De novo Afs formation was detected in grafts containing base-edited fibroblasts, whereas no fully formed Afs were observed in untreated grafts. Images are shown at the following magnifications: ×15,000, bar = 500 nm and ×30,000, bar = 200 nm. Green arrows point at Afs. WT KC/FBs denotes wild-type grafts, Unt RDEB KC/Unt RDEB FB denotes untreated RDEB grafts, and Unt RDEB KC/coCBE FB denotes grafts containing base-edited fibroblasts and untreated RDEB keratinocytes. AF, anchoring fibril; C7, type 7 collagen; DEJ, dermal−epidermal junction; FB, fibroblast; KC, keratinocyte; RDEB, recessive dystrophic epidermolysis bullosa; TEM, transmission electron microscopy; WT, wild type.

The restoration of the integrity of the DEJ in the grafts was further confirmed by immunofluorescent analysis of C7 protein expression. No C7 was detected in the untreated RDEB grafts, whereas deposition of the protein at the DEJ was observed in the grafts containing base-edited fibroblasts (Figure 7a and Figure 8a and c).

Importantly, transmission electron microscopy was used to assess whether C7 expression in the grafts containing base-edited fibroblasts translated to de novo AF formation. AFs were quantified by a well-established quantitative ultrastructural technique in which AFs were counted along a 40-μm continuous stretch of the DEJ in a blinded fashion (Tidman and Eady, 1985). A blistering phenotype and extensive dermal−epidermal separation were observed in all RDEB grafts, with only 15 μm of unseparated skin available for evaluation in two of the three samples, with mostly wispy, rudimentary AFs seen (Figure 7b and Figure 8b and d). On the contrary, the micrographs of the grafts containing base-edited fibroblasts revealed an abundance of sublamina densa fibrillary structures that bore the ultrastructural characteristics of normal Afs, exhibiting cross-banding and extending ∼200 nm into the dermis, looping around type I and III dermal collagen fibers (Figure 7b). No blistering or tissue cleavage was observed at the DEJ, consistent with functional restoration of dermal−epidermal adhesion. AF quantification confirmed a significantly (n = 3, *P* = 0.0002) higher number of AFs in the grafts containing base-edited fibroblasts than in the untreated RDEB grafts (Figure 7c). These data clearly show that the base-editing strategy not only led to the restoration of C7 expression in vivo but also conferred functional correction of the DEJ through the formation of de novo AFs.

## Discussion

This study investigated the potential of an early-generation CBE to correct the known recurrent c.425A>G mutation within *COL7A1*. This splice-site mutation at position −2 at the donor splice site of exon 3 causes aberrant splicing of at least two abnormal transcripts, leading to a premature termination codon downstream in the *COL7A1* gene (Gardella et al., 1996; Hammami-Hauasli et al., 1997). Because of the proximity of the gene start, C7 protein expression and hence AFs are completely absent in patients homozygous for this mutation, often presenting with severe RDEB.

Genome editing promises to overcome the limitations of conventional gene addition approaches, especially for large transgenes. For RDEB-causative mutations, canonical CRISPR/Cas9-mediated correction through homology-directed repair relies on the efficient delivery of donor repair templates. Template delivery includes nonviral methods, including plasmid DNA (Hainzl et al., 2017), double-stranded DNA (Webber et al., 2016), single-stranded oligonucleotides (Jacków et al., 2019), or viral-based delivery (Izmiryan et al., 2018; Osborn et al., 2020).

Base editors, on the other hand, do not rely on the homology-directed repair pathway and hence alleviate the need for donor template delivery for the correction of single nucleotide mutations. Furthermore, base editors delivered as mRNA exhibit transient expression and have a reduced risk of aberrant effects (Koblan et al., 2018; Rees and Liu, 2018). Recently, adenine base editor delivery within virus-like retroviral particles was used to correct an RDEB mutation (Osborn et al., 2020), further showing the adaptability of the platforms.

In our experiments, an early generation base editor, CBE3, combined D10A Cas9 nickase with rat APOBEC1 (rAPOBEC1) cytidine deaminase. This converts cytosine into uracil within a 5-bp catalytic window of activity between the fourth and eighth bases distal to the PAM on the nontarget strand of the sgRNA (Komor et al., 2016). Uracil is subsequently converted to thymine during DNA replication or repair, whereas the inclusion of an inhibitor of uracil DNA glycosylase prevents base excision repair. Subsequent iterations have employed additional uracil DNA glycosylase elements and improved fidelity to reduce the likelihood of indel creation, off-target effects, and RNA deamination.

The c.425A>G mutation was amenable for CBE-mediated conversion given that a pathogenic substitution is located at position 5 of the base-editing window, albeit with an adjacent cytosine nucleotide. Codelivery of the sgRNA and *CoBE3* mRNA into primary fibroblasts and patient-derived iPSCs resulted in on-target conversion rates of 61 and 45%, respectively. These results were confirmed through deep sequencing by NGS, where >59% and 51% of the targeted c.425A>G mutation correction was detected in patient iPSCs and fibroblasts, respectively. Importantly, bystander edits were detected at position C4 in 4.8 and 19.4% of NGS reads in patient fibroblasts and iPSCs, respectively. Computational predictions of C>T changes indicated by bystander edits at this position alone or in combination with the corrective edit may result in splicing aberrations involving partial or total exon 3 skipping and/or activation of a cryptic splice donor site because of the alteration of a splice donor sequence in exon 3, similar to splicing aberrations previously reported in patients with c.425A>G (Gardella et al., 1996). Importantly, NGS-based haplotype analysis of sequencing reads from patient fibroblasts confirmed that up to 46% of the cells contained the corrective C>T edit at the desired position alone, without unwanted on-target events.

Overall, bystander edits, both within and outside the editing window, were more frequent in patient iPSCs than in fibroblasts. In agreement with previous studies (Komor et al., 2016), C-to-non-T edits were also detected at low frequencies. As mentioned earlier, these effects may be addressed by next-generation base editors with higher editing fidelity and specificity (Kim et al., 2017; Komor et al., 2017; Ma et al., 2016).

Off-target edits were also investigated by deep sequencing, and no appreciable base-editing activity was observed in 9 of 10 in silico predicted sites. The 4% substitution frequency at C5 in off-target 3 was present in controls and likely a naturally arising variant in cultured cells. It is worth noting that in silico off-target detection tools have predictive limitations (Chuai et al., 2018; Wilson et al., 2018). Unbiased genome-wide approaches include in vitro cell-based methods with high-throughput sequencing of genomic DNA (Doman et al., 2020) and include Digenome-seq (digested genome sequencing) (Kim et al., 2015). However, the generation of CBE protein required for such examinations has proven problematic, and assays screening for Cas9 nuclease effects (Cameron et al., 2017; Tsai et al., 2017, 2015) have limited relevance. Nevertheless, NHEJ activity and indel formation due to the nicking of the nonedited strand is an important consideration, with 3.5% NHEJ activity detected in on-target amplicons. Again, this is likely to be addressed by the inclusion of additional uracil DNA glycosylase elements in next-generation editors. Our in silico predicted sites from Benchling were corroborated using the CRISPR RGEN Cas-OFFinder (Bae et al., 2014), CRISPRoff (Alkan et al., 2018), and CRISPOR (Concordet and Haeussler, 2018) algorithms. We were able to cross-verify 8 of 10 of the sites interrogated by NGS across the four platforms. This type of predicted off-target analysis has quite major limitations and provides only a rudimentary analysis of guide-dependent effects, without accounting for guide-independent or promiscuous activity. Furthermore, RNA targeting by cytidine deaminases has also been described (Grünewald et al., 2019), albeit after transfection of base editor plasmids into a human embryonic kidney 293T cell line. Delivery of the codon-optimized base editor (coCBE3) in the form of mRNA, as described in this study, may mitigate such concerns as a result of its transient expression within the cells. We have previously interrogated the possibility of promiscuous guide-independent C>N deamination of antigen-specific receptor RNA collected from serial samples taken from primary human T cells edited with coCBE3, with no evidence of RNA deamination compared with that of the controls (Georgiadis et al., 2021; Preece et al., 2020).

Restoration of C7 protein expression in base-edited fibroblasts was confirmed by immunofluorescence microscopy and western blotting. Importantly, western blotting using cell culture supernatant revealed the presence of full-length C7, indicating successful secretion of the protein. This translated to the deposition of functional proteins at the DEJ in vivo and the formation of de novo AFs. Although C7 deposition was not continuous along the basement membrane zone, with patches devoid of the immunofluorescent signal observed, ultrastructural analysis confirmed that the grafts engineered using base-edited fibroblasts contained a significantly higher number of AFs than untreated RDEB grafts, where extensive dermal−epidermal separation and blistering were observed. Although the number of AFs in grafts containing base-edited fibroblasts was approximately half that detected in WT control grafts, this amount was sufficient to effectively repair epidermal−dermal adhesion and restore skin functionality. Previously, it has been shown that approximately 10% of WT C7 levels are required for AF formation and significant phenotype improvement in hypomorphic RDEB mouse models (Nyström et al., 2013). A recent study showed that skin equivalents composed of 11 and 15% CRISPR/Cas9-gene corrected KCs and fibroblasts, respectively, resulted in 26% C7 re-expression and AF formation in vivo (Izmiryan et al., 2018).

Importantly, we were also able to successfully edit patient-derived iPSCs and show the restoration of protein expression in iPSC-derived KC-like cells by immunofluorescence. Because only a limited number of patient cells can be obtained from the skin biopsies of patients with RDEB, iPSCs may in the future provide a source of material for autologous transplantation of therapeutically relevant cells, including fibroblasts, KCs, and MSCs (Itoh et al., 2013; Jacków et al., 2019; Webber et al., 2016).

Another recent study has shown the feasibility of adenosine base editors for the correction of two RDEB causative mutations (Osborn et al., 2020). C7 protein restoration was confirmed in a three-dimensional skin culture model in vitro and in a teratoma assay in vivo, where base-edited iPSCs formed epithelial-like structures. However, C7 expression or AF formation was not examined in the skin using a humanized mice model, as described in this study.

Overall, this report adds to the evidence of the feasibility of base-editing technology to correct *COL7A1* mutations and restore skin functionality through the formation of de novo AFs but also highlights the limitations of early-generation base-editing tools. Ongoing improvements to narrow the base-editing window, eliminate residual cleaving activity, and minimize promiscuity may address these issues and provide novel therapeutic avenues for RDEB.

## Materials and Methods

### Isolation and culture of primary fibroblasts

Skin biopsies were obtained with authorization from the National Research Ethics Services, Westminster (07/H0802/104) and written informed consent. Fibroblasts homozygous for the c.425A>G mutation were isolated as previously described (Georgiadis et al., 2016) and cultured in DMEM supplemented with 10% fetal bovine serum and 1% penicillin−streptomycin.

### Reprograming of primary fibroblasts to iPSCs

Patient iPSC lines were generated using the CytoTune iPS 2.0 Sendai Reprogramming (Thermo Fisher Scientific, Waltham, MA) under feeder-free conditions. The resultant colonies were cultured in TESR2 (STEMCELLS Technologies, Vancouver, Canada) on laminin-511−coated plates (Biolamina, Sundbyberg, Sweden) at a concentration of 2.4 μg/ml.

### iPSC characterization

Antibodies used for iPSCs characterization are listed in Table 2. For in situ immunofluorescence, cells were seeded onto sterile 13 mm coverslips in a 24-well plate, cultured for 48 hours, then fixed in 4% paraformaldehyde, blocked and permeabilized with 0.1% Triton X-100 and 3% BSA, and then incubated overnight at 4 °C with the primary antibody, followed by incubation with the secondary antibody and counterstaining with DAPI. The coverslips were mounted on microscope glass with Prolong gold. Micrographs were imaged using a Zeiss Observer 7 (Zeiss, Oberkochen, Germany) and processed using ImageJ (Wayne Rasband [National Institutes of Health, Bethesda, MD]).

**Table 2:** List of Antibodies Used in this Study

Target	Company	Application
Monoclonal Anti-Collagen Type VII, LH7.2 clone	Sigma-Aldrich	Immunofluorescence
Polyclonal Anti-Collagen Type VII	Gift from Prof Chen	Immunoblotting
Polyclonal Anti-Keratin 14	BioLegend	Immunofluorescence
Monoclonal Anti-Keratin 10	Abcam	Immunofluorescence
Monoclonal Anti-Human Cytochrome C oxidase (Complex IV) subunit II	Abcam	Immunofluorescence
Monoclonal Anti-Vinculin	Sigma-Aldrich	Immunoblotting
Anti-SOX2-human-FITC	Miltenyi Biotech	Flow cytometry
Anti NANOG-human APC	Miltenyi Biotech	Flow cytometry
Anti TRA 1-60-human PE	Miltenyi Biotech	Flow cytometry
Anti TRA 1-81-human PE	Miltenyi Biotech	Flow cytometry
Anti SSEA-4-human PE	Miltenyi Biotech	Flow cytometry
Polyclonal Anti-SOX2	Sigma-Aldrich	Immunofluorescence
Monoclonal Anti-OCT-3/4	Santa Cruz Biotech	Immunofluorescence
Monoclonal Anti-AFP	Sigma-Aldrich	Immunofluorescence
Monoclonal Anti-ACTA2	Sigma-Aldrich	Immunofluorescence
Monoclonal Anti-TUBB3	Sigma-Aldrich	Immunofluorescence
Monoclonal Anti-ΔNp63	Abcam	Immunofluorescence

Abbreviations: APC, allophycocyanin; PE, phycoerythrin.

For iPSC characterization by flow cytometry, cells were incubated with the antibody at 4 °C for 20 minutes for extracellular markers or were fixed in Fix & Perm Medium A (Thermo Fisher Scientific) for 20 minutes at room temperature, followed by incubation with the antibody at 4 °C for 1 hour in Fix & Perm Medium B for intracellular markers. Cell acquisition was carried out on a 2-laser CyAn ADP Analyzer.

For pluripotency assessment at the cDNA level, total RNA was extracted using the RNeasy Mini Kit (Qiagen, Hilden, Germany) and retrotranscribed, using the High-Capacity cDNA Reverse Transcription Kit (Thermo Fisher Scientific) according to the manufacturer’s instructions before RT-PCR amplification.

### Trilineage differentiation

To make embryoid bodies, undifferentiated cells were dissociated as single cells (day 0) with Accutase (Thermo Fisher Scientific) for 8 minutes at 37 °C and seeded at high density in AggreWell800 (STEMCELLS Technologies). Cells were resuspended in EB formation medium (STEMCELLS Technologies) supplemented with 10 μM of HA-100 (STEMCELLS Technologies) for 1 week (day 7). iPSC aggregates were then transferred on Matrigel-coated plates with coverslips and cultured in DMEM 10% fetal bovine serum for 3 weeks. After differentiation, cells were fixed in 4% paraformaldehyde and analyzed by immunofluorescence for the expression of mesoderm, endoderm, and ectoderm markers.

### Directed differentiation of iPSCs into KCs

Base-edited iPSCs were differentiated into KCs as previously described (Petrova et al., 2014).

### CBE3-mediated base editing

The ×3C7-CyD sgRNA (CACCCTGGGGACACCAGGTC, antisense orientation) was designed using the online Benchling CRISPR design tool (https://benchling.com/crispr). Synthetic sgRNAs were manufactured by Synthego (Redwood City, CA) using automated solid-phase synthesis with 2’-O-methyl 3’ phosphorothioate modifications in the first and last three nucleotides. A third-generation CBE (CBE3) plasmid was human codon optimized, and mRNA was custom synthesized by TriLink using proprietary CleanCap technology to increase expression and stability. For the delivery of base-editing reagents, 1 × 10^6^ cells were electroporated with 2 μg of sgRNA and 5 μg of *coCBE3* mRNA in 100 μl cuvettes of 4D-Nucleofector X Unit using EN-150 or CA-137 program for fibroblasts and iPSCs, respectively. Cells were then cultured at 30 °C for 24 hours before returning to 37 °C culture conditions.

### Assessment of CBE3-mediated activity

Seven days after electroporation, DNA was extracted using DNeasy Blood & Tissue Kit (Qiagen) and PCR across the target site (exon 5 *COL7A1* reverse GGAACTCACGAGGTCGGGTC and intron 2 *COL7A1* forward CAGTGCAGTACAGCGATGACC) was performed using Q5 High-Fidelity DNA Polymerase Master Mix (New England Biolabs, Ipswich, MA). Purified PCR products were analyzed using Sanger sequencing−based EditR analysis.

### NGS for the assessment of on- and off-target events

Online software, Benchling, was used to predict the top 10 in silico off-targets for the designed guide sequence (Table 3). NGS libraries for on- and off-target sites were prepared using a Nextera XT Kit (Illumina, Cambridge, United Kingdom). Products were amplified using combinations of target-specific primers (Table 4). After the library preparation, individually barcoded samples were pooled and run in MiSeq using a 500-V2 nano-cartridge. Demultiplexed fastq files were uploaded to Galaxy (Afgan et al., 2018) for quality check, trimming, and alignment. Base conversions and NHEJ signatures were analyzed using Naïve Variant Caller and Pindel, respectively (Ye et al., 2009). Figures were created using GraphPad Prism (San Diego, CA).

**Table 3:** Top 10 In Silico Predicted Sites Interrogated by NGS

Off-Target	Sequence	PAM	Chromosome	Strand	Position
C7-ON	CACCCTGGGGACACCAGGTC	GGG	chr3	−1	48593551
C7-OT1	TACCCTGGGGGCACCAGGTC	CAG	chr10	−1	71798536
C7-OT2	CACCCTGGAGACACCAGGAC	TAG	chr19	−1	19637825
C7-OT3	GACCCTGGGTACACCAGGTC	AGG	chr5	1	65716931
C7-OT4	CACCCTGGGGACAGCAGGTA	GGG	chr6	1	161111959
C7-OT5	CACCCTGGGGACAGCATGTC	CAG	chr16	1	88952889
C7-OT6	GAGCCTGGGGACACCAGGTG	CAG	chr12	−1	5873281
C7-OT7	GACCCTGGGGCCACCAGGCC	AGG	chr7	1	149729342
C7-OT8	AACCCTGGGAACACCAGGCC	AAG	chr17	1	31512778
C7-OT9	CTCCCTGGGGTCACCAGGCC	GAG	chr17	1	76982342
C7-OT10	CTCCCTGGGGACATCAGGGC	TGG	chr1	−1	6337136

Abbreviations: chr, chromosome; PAM, protospacer adjacent motif.

**Table 4:** Primer Sequences for NGS Library Preparation

Primer Name	Sequence
C7-ON FWD	CGGTTCCCCTGGACACTT
C7-ON REV	ACAGGACAGAGTTCGGCC
C7-OT1 FWD	TACGCCCCAGTTCAAGCC
C7-OT1 REV	AGGGGCTGTGGTCTCTCT
C7-OT2 FWD	AGGCATGGTCAGAGCAGG
C7-OT2 REV	CCAAGCAGCGAATCGTGT
C7-OT3 FWD	AAAGGTCTGGGCTGAGGG
C7-OT3 REV	TGGTCAGTTCTCAGCTTTCAT
C7-OT4 FWD	AATGCCCAGACCATGCCT
C7-OT4 REV	AGCCCAAGTGTGTGAGGA
C7-OT5 FWD	CCCCATGACAGCCCATCA
C7-OT5 REV	TCAGCAGCAAACCCGATG
C7-OT6 FWD	GAGTGAGGGCTGAGCAGT
C7-OT6 REV	TTGCCCACAGAGTCCCAG
C7-OT7 FWD	CAGGACTGAGGGCTGAGG
C7-OT7 REV	GTCAGTACCGAGGGCAGG
C7-OT8 FWD	GGCTCTGGGTCTTGAGGG
C7-OT8 REV	CCAGGGCAGCTTCCAAGA
C7-OT9 FWD	ACAGAGAGGCAGCCGAAG
C7-OT9 REV	CTGCTTCCCCTGCCAGAA
C7-OT10 FWD	TCCTGCCTTCTCCAAGCC
C7-OT10 REV	AGCATGAGAGAGCAGCCC

Abbreviations: FWD, forward; NGS, next-generation sequencing; REV, reverse.

Primers are given in the 5´−3´ orientation.

### Immunofluorescence and immunoblotting

Immunofluorescence and immunoblotting were performed as previously described (Petrova et al., 2020). Immunofluorescent detection was performed with a monoclonal C7 antibody (LH7.2 clone, Sigma-Aldrich, St Louis, MO), whereas a polyclonal anti-human C7 antibody (Chen et al., 2002) was used for immunoblotting (Chen et al., 2002). A full list of antibodies used in this study is available in Table 2. Staining was visualized and imaged using a Zeiss Observer 7 and processed using ZEN Pro software (Zeiss). Postprocessing and quantification were carried out using Fiji as described earlier (Petrova et al., 2020). For immunoblotting, the total protein concentration was determined using Pierce 660nm Protein Assay (Thermo Fisher Scientific), and equal quantities (65 μg) of total protein were loaded on SDS-PAGE.

### Bioengineered skin preparation and grafting on immunodeficient mice

The methods for preparing and grafting bioengineered skin on immunodeficient NOD-scid IL2Rgammanull mice have been described previously (Petrova et al., 2020). In brief, for the dermal compartment, 1.5 × 10^5^ WT fibroblasts were used, untreated RDEB ([+/+] c.425A>G, p.Lys142Arg) fibroblasts or base edited RDEB fibroblasts ([+/+]) (n = 3 each). WT or RDEB KCs were used for the epidermal compartment for WT or RDEB (both containing untreated and base-edited fibroblasts) grafts, respectively. All animal studies were approved by the University College London Biological Services Ethical Review Committee and licensed under the Animals (Scientific Procedures) Act 1986 (Home Office, London, United Kingdom).

### Transmission electron microscopy

Sample processing for transmission electron microscopy was performed as previously described (Georgiadis et al., 2016). Images were acquired with a JEOL JEM 1400 Plus transmission electron microscopy with a JEOL Ruby CCD camera (JEOL, Welwyn Garden City, United Kingdom). Consecutive 40 overlapping images covering 40 μm of a well-defined lamina densa were taken at ×15,000 magnification in a blinded fashion, where the identity of the samples was unknown during imaging, and AF quantification and unblinded only after those were completed. AF scoring was performed using established quantitative ultrastructural techniques (Tidman and Eady, 1985). Student’s *t*-test was used to carry out the statistical analysis.

### Data availability statement

The next-generation sequencing dataset related to this article can be found at https://submit.ncbi.nlm.nih.gov/subs/bioproject, hosted at the BioProject National Center for Biotechnology Information repository. BioProject identification is PRJNA906066. Other datasets necessary to interpret and/or replicate the data in this paper are available upon request to the corresponding author.

## ORCIDs

Gaetano Naso: http://orcid.org/0000-0002-7664-7260

Soragia Athina Gkazi: http://orcid.org/0000-0002-6385-368X

Christos Georgiadis: http://orcid.org/0000-0003-0031-8396

Vignesh Jayarajan: http://orcid.org/0000-0002-1185-3274

Joanna Jacków: http://orcid.org/0000-0002-5744-7896

Roland Fleck: http://orcid.org/0000-0003-1542-6218

Leanne Allison: http://orcid.org/0000-0003-2954-9892

Olumide Kayode Ogunbiyi: http://orcid.org/0000-0001-5208-5526

John Alexander McGrath: http://orcid.org/0000-0002-3708-9964

Dusko Ilic: http://orcid.org/0000-0003-1647-0026

Wei-Li Di: http://orcid.org/0000-0002-4851-1649

Anastasia Petrova: http://orcid.org/0000-0001-5294-399X

Waseem Qasim: http://orcid.org/0000-0001-8353-4494

### Disclaimer

The views expressed are those of the authors and not necessarily those of the NHS, the National Institute for Health and Care Research, or the Department of Health.

## Conflict of Interest

The authors state no conflict of interest.
